# Structural Characterization and Anti-Proliferation Activities Against Tumor Cells of an Arabinogalactan from *Juniperus convallium*

**DOI:** 10.3390/molecules24101850

**Published:** 2019-05-14

**Authors:** Beibei Li, Mengxue Dong, Ji De, Li Ye, Daofeng Chen, Yan Lu

**Affiliations:** 1 School of Pharmacy, Fudan University, Shanghai 201203, China; 16211030026@fudan.edu.cn (B.L.); 16211030047@fudan.edu.cn (M.D.); yelil@fudan.edu.cn (L.Y.); dfchen@shmu.edu.cn (D.C.); 2 College of Science, Tibet University, Lhasa 850000, Tibet, China; 12110700018@fudan.edu.cn

**Keywords:** *Juniperus convallium*, polysaccharide, structure characterization, proliferation, antitumor activities

## Abstract

As a hyperproliferative disorder, cancer has continued to be a major public health challenge. In the present study, a polysaccharide JC-PS1 was isolated and purified from *Juniperus convallium*. JC-PS1 is a heteropolysaccharide composed of Ara, Gal, GalA and Rha with the average molecular weight of 280 kDa. Based on the methylation and 2D NMR analysis, JC-PS1 was elucidated as a backbone of →5)-α-Ara*f*-(1→ and →3,5)-α-Ara*f*-(1→, and three kinds of branches attached to the O-3 position of →3,5)-α-Ara*f*-(1→, including β-Gal*p*A-(1→3)-β-Gal*p*-(1→, α-Ara*f*-(1→3)-α-Rha*p*-(1→ and α-Ara*f*-(1→3)-β-Gal*p*-(1→. Accordingly, the atomic force microscopy of JC-PS1 showed a linear filamentous structure with small proportion of branches. Furthermore, JC-PS1 exhibited significant anti-proliferation activities against PANC-1, A431, MDA-MB-231, U118MG and H1975 cells with the IC_50_ values of 296.8, 477.9, 657.4, 686.7 and 862.1 μg/mL, respectively. This indicated that JC-PS1 could be a potential therapeutic agent for the treatment of cancer.

## 1. Introduction

As a hyperproliferative disorder, cancer has continued to be a major public health challenge and is still the leading cause of morbidity and mortality all over the world [1]. The current therapeutic strategies have remained to be systemic chemotherapy and radiotherapy after surgeries in the last few years. However, the prognosis still remains poor because of drug resistance and severe side effects [2]. It is of great necessity to discover new safe and effective anti-cancer agents.

Polysaccharides, as a kind of biological macromolecules, have aroused more and more attention in the past ten years because of their relatively low toxicity and diverse biological activities, such as antitumor [3,4], antidiabetic [5], hypoglycemic [6,7], antiviral and immune regulatory effects [8]. In particular, natural polysaccharides have become a promising and valuable source of therapeutic agents for the treatment of cancer recently. More and more reports have pointed out that natural polysaccharides had significant anti-proliferation activities against various tumor cell lines of human origin such as HepG2, SiHa, MDA-MB-231, A549, KB, BXCP-3, SGC-7901, Hela, MCF-7 cells [9,10,11], and could induce apoptosis in them [12,13].

*Juniperus* species (Cupressaceae), which belong to conifers and consist of 75 species worldwide, are a good bet in the development of new drugs with natural compounds, especially antitumor drugs [14]. Small molecules from *Juniperus* species have been reported to show significant cytotoxic potential for tumor cells. For example, the n-hexane extracts from *J. phoenicea* showed broad spectrum of anti-proliferation activities against HepG2, MCF-7 and A549 cells [15]. In addition, the essential oil of *J. phoenicea* var. *turbinata* (syn. *J. turbinata* Guss.) exhibited cytotoxic effects against HCT116, A375, and MDA-MB-231 cells [16]. In addition, deoxypodophyllotoxin, an aryltetralin cyclolignan which is characterized of the cytotoxic potential [17,18], has been isolated from several *Juniperus* species like *J. virginiana*, *J. rigida*, *J. sabina*, *J. squamata*, *J. procera*, *J. bermudiana*, *J. chinensis*, and so on [19]. However, the antitumor activities of polysaccharides from *Juniperus* species have not been studied and discussed yet.

*J. convallium* Rehder & E. H. Wilson., a *Juniperus* species peculiar to China, is mainly distributed in the south of Qinghai, northwest of Sichuan and east of Tibet at altitudes of 2200 m to 4300 m. In the present study, we report the isolation and structural characterization of a polysaccharide, JC-PS1, which was isolated and purified from *J. convallium*, as well as its structural characterization and antitumor activities against various human cancer cells.

## 2. Results and Discussion

### 2.1. Homogeneity and the Relative Molecular Weight of JC-PS1

**Figure 1:**
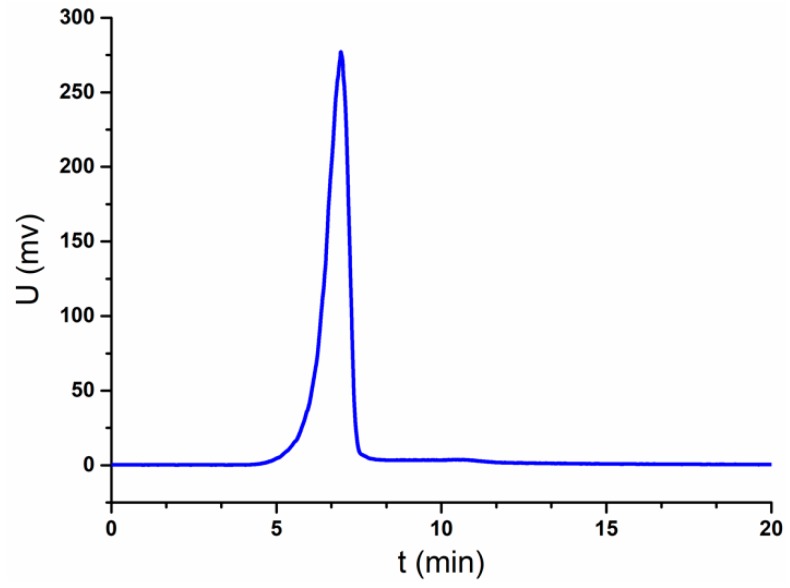
HPGPC-ELSD chromatogram of JC-PS1.

JC-PS1 was obtained from the crude polysaccharides extracted from the twigs and leaves of J. convallium by using anion-exchange chromatography DEAE-52 and Sephacryl S-300 gel filtration column. The homogeneity of JC-PS1 was identified by HPGPC equipped with evaporative light scattering detector (ELSD). The HPGPC-ELSD chromatogram exhibited a single and symmetrical peak at 6.953 min in Figure 1, suggesting that JC-PS1 was a homogenous polysaccharide. The relative molecular weight of JC-PS1 was also determined by HPGPC-ELSD with the standard curve method with a series of dextran with different molecular weights as standards. According to the retention time of JC-PS1and the standard curve, the relative molecular weight of JC-PS1 was calculated as 280 kDa.

### 2.2. Chemical Composition Analysis

**Figure 2:**
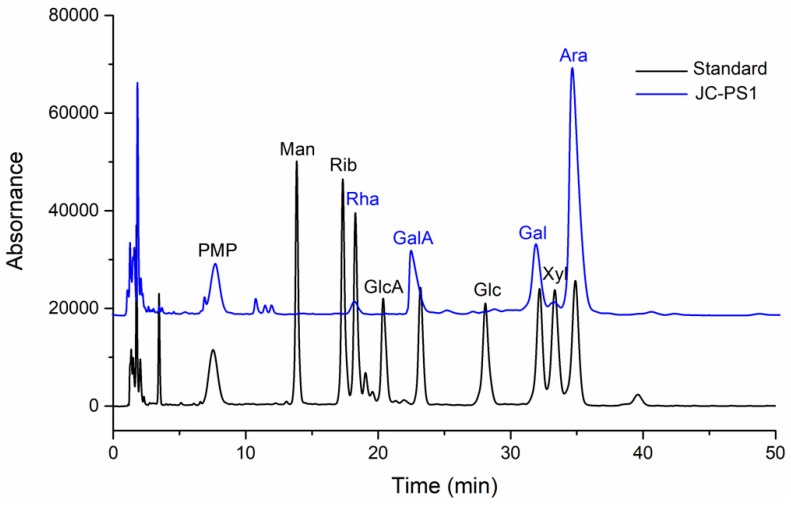
The PMP-LC-MS chromatogram of monosaccharide composition analysis of JC-PS1.

The chemical composition of JC-PS1 was firstly analyzed in order to clearly illuminate the structure. The total carbohydrate content of JC-PS1 was 98.5%. The content of uronic acid and proteins of JC-PS1 was 12.1% and 1.2%, respectively. In addition, then the 1-phenyl-3-methyl-5-pyrazolone (PMP) pre-column derivatization was conducted to analyze the composition and content of monosaccharide in JC-PS1. The results (Figure 2) indicated that JC-PS was a heteropolysaccharide composed of arabinose (Ara), galactose (Gal), galacturonic acid (GalA) and rhamnose (Rha) with molar ratio of 66.9:17.8:11.2:4.1, which suggested that the arabinose was the main backbone of JC-PS1.

### 2.3. FT-IR Spectrum Analysis of JC-PS1

FT-IR spectrum of carbohydrates could provide information on the structural features. The FT-IR spectrum of JC-PS1 is shown in Figure S1. The absorption bands at 3400 cm^−1^, 2930 cm^−1^, 1641 cm^−1^, 1040 cm^−1^ were characteristic absorption peaks of carbohydrates. The broad absorption peaks at 3400 cm^−1^ and 2930 cm^−1^ were attributed to the O-H stretching vibrations and C-H stretching vibrations, respectively [20]. The bands at 1641 cm^−1^ and 1425 cm^−1^ were due to the asymmetric and symmetric COO- stretching vibrations and assigned to the existence of uronic acid in JC-PS1 [21,22], which was in accordance with the results of the monosaccharide composition analysis. The absorption bands at 1140 cm^−1^ and 1072 cm^−1^ indicated the existence of a pyranose configuration in JC-PS1 [23]. The absorption at 890 cm^−1^ indicated a β-configuration in JC-PS1 [24].

### 2.4. Methylation Analysis of JC-PS1

Methylation analysis is an indispensable method in the structure characterization of carbohydrates, from which the linkage types in carbohydrates can be further identified. After the reduction of the galacturonic acid in JC-PS1, the reduced JC-PS1 was then methylated three times and the fully methylated JC-PS1 was hydrolyzed and derived into the corresponding alditol acetate derivatives, which were further analyzed by GC-MS. Compared to the MS data in the CCRC database (https://www.ccrc.uga.edu/specdb/ms/pmaa/pframe.html), the GC-MS results revealed that there were six linkage types existing in JC-PS1, which are listed in Table 1. The Ara residues existed in the form of furanose, including 1-linked, 1,5-linked and 1,3,5-linked Araf residues. The Rha residues existed in the form of pyranose, 1,3-linked Rhap residues. Because the corresponding alditol acetate derivative of the reduced GalA was the same with that of Gal, the linkage types of the GalA and Gal residues couldn’t be concluded directly. However, we found that the molar ratios of 1-linked Galp residues (10%) and 1,3-linked Galp (15%) were roughly equal to that of the GalA (11.2%) and Gal (17.8%) in the analysis of monosaccharide composition, respectively, so we speculated that the GalA residues might existed in 1-linked GalpA, and the Gal residues in 1,3-linked Galp.

To sum up, there were six linkage types in JC-PS1, 1,5-linked Araf, 1-linked Araf, 1,3,5-linked Araf, 1,3-linked Glap, 1,3-linked Rhap and 1-linked GalpA. In addition, the molar ratio was 3.9:1.0:2.1:1.5:0.5:1.0, which approximately agreed with the results of monosaccharide composition and indicated that 1,5-linked Araf might be the backbone of JC-PS1. However, it is necessary to confirm the detailed information by the NMR spectra analysis.

### 2.5. NMR Spectra Analysis of JC-PS1

To further elucidate the structure of the JC-PS1, NMR spectroscopy, an efficient and convenient technique, was used to provide more straightforward and detailed information on the structure of JC-PS1, including anomeric configurations, linkage patterns and linkage sequences of the repeating units. Based on methylation analysis, monosaccharide composition analysis and NMR analysis, their chemical shifts were assigned as listed in Table 2.

**Figure 3:**
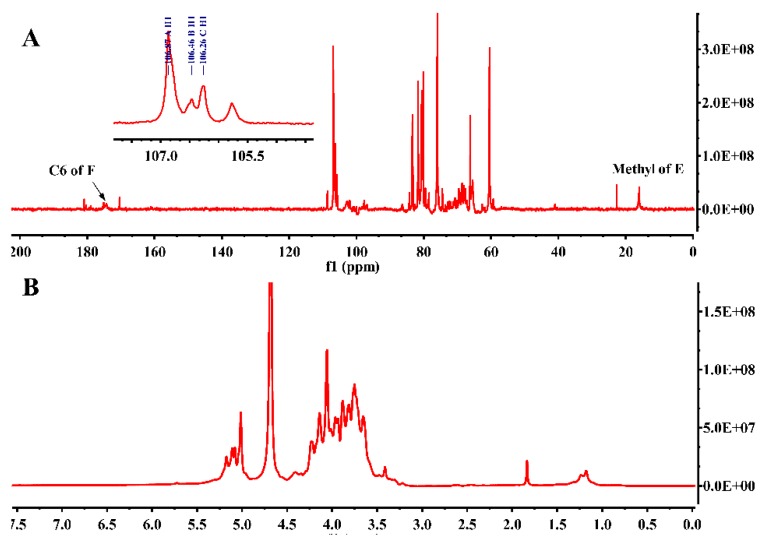
The ^13^C NMR spectrum (**A**) and ^1^H NMR spectrum (**B**) of JC-PS1.

**Figure 4:**
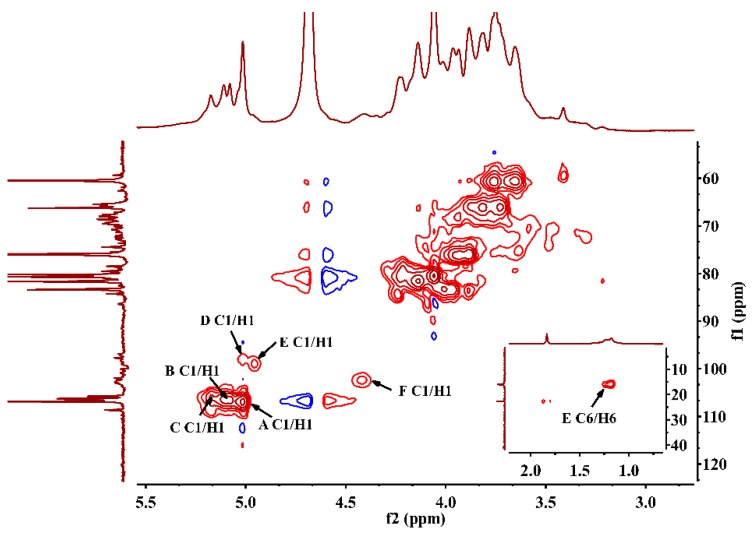
HSQC spectrum of JC-PS1.

In the ^13^C NMR spectrum (Figure 3A), anomeric carbon signals were presented at around δ 98–107 ppm. In addition, the downfield chemical shifts at δ 106.93, 106.51, 106.05 ppm were the characteristic signals of arabinosyl present in furanose form [25], which were designated to be the C-1 of residue A-C. Comparing the integrated peak areas of ^13^C spectrum and the molar ratio of the Ara residues in the methylation analysis, the residues A-C were identified as 1,5-linked Ara*f*, 1-linked Ara*f* and 1,3,5-linked Ara*f*. In addition, the corresponding anomeric proton signals were presented at δ 5.02 ppm (A H1), 5.10 ppm (B H1), 5.17 ppm (C H1) according to HSQC (Figure 4), which suggested that they belonged to α-anomeric configuration with low-field signals (>5.0 ppm) [26]. For residue A, according to ^1^H-^1^H COSY spectrum (Figure S2), we correlated A H1 (5.02 ppm) and A H2 (4.06 ppm), A H2 (4.06 ppm) and A H3 (3.93 ppm), A H3 (3.93 ppm) and A H4 (4.14 ppm), A H4 (4.14 ppm) and A H5 (3.73 ppm). In addition, the corresponding carbon signals were assigned as A C1-C5 (δ 106.93, 80.47, 76.21, 3.93, 4.14, 3.73 ppm) by HSQC spectrum. Similarly, the proton and carbon signals of residue B and C were assigned.

The signal at 98.96 ppm was assigned to C1 of 1,3-β-Gal*p* (residue D) [27]. The cross peak (4.96/98.96 ppm) in HSQC showed that the anomeric proton signal of residue D was assigned at 4.96 ppm. According to ^1^H-^1^H COSY and HSQC, the signals at δ 75.88, 78.51, 73,55, 68.64 and 60.61 ppm were assigned to C2-C6 of residue D, and the corresponding signals at δ 3.76, 3.59, 3.82, 3.93 and 3.65 ppm were assigned to H2-H6 of residue D.

The signal at 98.00 ppm was assigned to the C1 of 1,3-α-Rha (residue E) [28]. In addition, the signal at 5.02 ppm was attributed to the corresponding H1 of residue E according to HSQC. The characteristic carbon signal at 15.96 ppm belonged to -CH3 (C6) of residue E and the chemical shift of the corresponding H6 was assigned at 1.18 ppm. The signals at δ 80.37, 81.21, 66.10 and 66.05 ppm were assigned to C2-C5 of residue E, and the signals at δ 4.21, 3.82, 3.73 and 3.70 ppm were assigned to H2-H5 of residue E.

The signal at 102 ppm was assigned to the C1 of 1-β-Gal*p*A (residue F) and the signal at 4.42 ppm was attributed to H1 of residue F. The characteristic carbon signal at 175.04 ppm was due to –COOH (C6) of residue F. The signals at δ 68.55, 70.73, 71.46 and 74.43 ppm were assigned to C2-C5 of residue F, and the signals at δ 3.29, 3.49, 3.63 and 3.48 ppm were assigned to H2-H5 of residue F.

**Figure 5:**
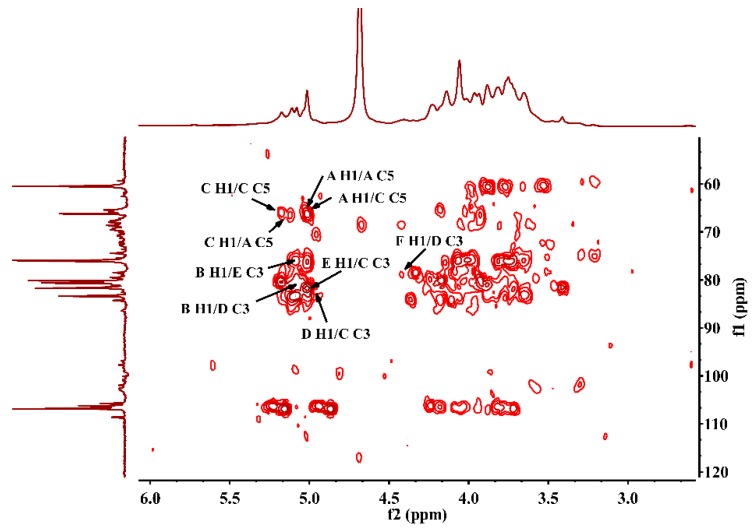
HMBC spectrum of JC-PS1.

After the assignment of the NMR signals and the identification of anomeric carbon configuration, the linkage sequences of glycoside residues were further elucidated by HMBC. The HMBC spectrum (Figure 5) showed the cross peaks between A H1 and A C5, A H1 and C C5,C H1 and C C5 and C H1 and A C5, indicating that self-connection and interconnection were present between residue A and C through 1→5 glycosidic bonds. Considering the molar ratio of the residues in methylation analysis, the backbone of JC-PS1 was indicated to be →5)-α-Ara*f*-(1→ (A) and →3,5)-α-Ara*f*-(1→ (C), and the branches were attached to the O-3 position of residue C. Then, the cross peaks between F H1 and D C3, D H1 and C C3 indicated that the terminal residue F was linked to residue D (F→D, R1), and were then attached together to the O-3 position of residue C as a branch. Similarly, the cross peaks between B H1 and E C3, E H1 and C C3 indicated that B→E (R2) was linked to O-3 position of residue C as a branch. In addition, the cross peaks between B H1 and D C3, D H1 and C C3 indicated that B→D (R3) was also a branch.

**Figure 6:**
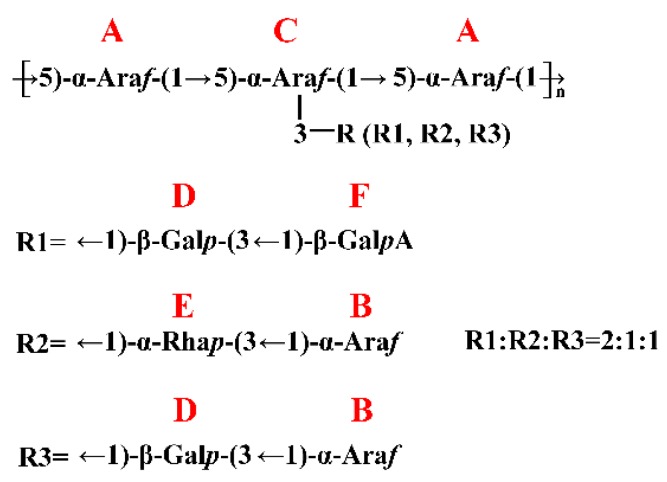
The repeating unit of JC-PS1.

Based on the results above, the backbone of JC-PS1 was indicated to be →5)-α-Ara*f*-(1→ and →3,5)-α-Ara*f*-(1→. In addition, three kinds of branches, F→D (R1), B→E (R2), and B→D (R3) were attached to O-3 position of →3,5)-α-Ara*f*-(1→. In addition, the molar ratio of the three branches (R1, R2, R3) was 2:1:1 according to the molar ratio of the residues in the methylation analysis. The repeating unit of JC-PS1 is shown in Figure 6.

### 2.6. Air Force Microscopy (AFM) Analysis of JC-PS1

**Figure 7:**
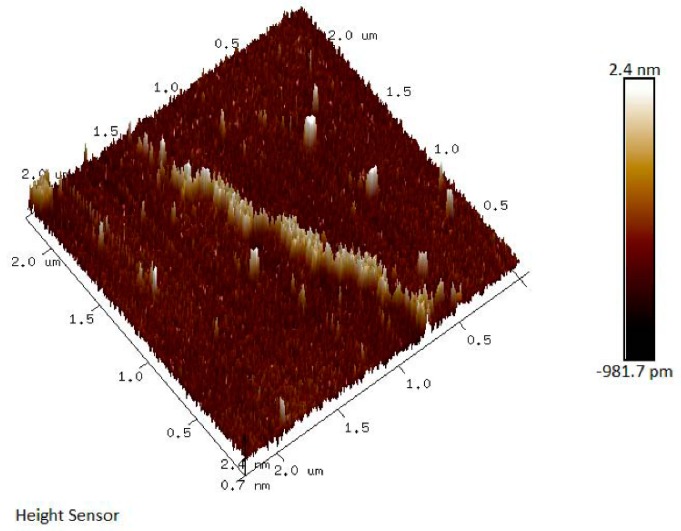
The AFM image of JC-PS1.

AFM could be applied to study the surface topography of polymers and biomacromolecules, which has aroused more and more attention [29]. The AFM image of JC-PS1 showed a linear filamentous structure with small proportion of branches as shown in Figure 7. In addition, the height and width of the structure was measured as 69.64 nm and 1.05 nm. In addition, the results were in accordance with the NMR and methylation analysis.

### 2.7. Anti-Proliferation Activities of JC-PS1

**Figure 8:**
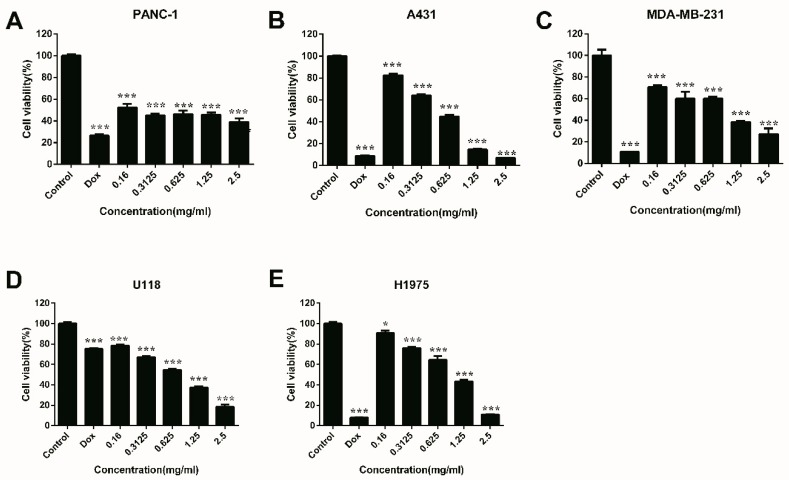
Anti-proliferation effects of JC-PS1 against PANC-1 (**A**), A431 (**B**), MDA-MB-231 (**C**), U118MG (**D**) and H1975 cells (**E**) with DOX as a positive reference. The data were represented as mean ± SD for triplicate, * *p* < 0.05; ** *p* < 0.01; *** *p* < 0.001; using the *t*-test.

Five carcinoma cell lines were chosen to test the anti-proliferation activities of JC-PS1, including PANC-1 pancreatic carcinoma cells, A431 human epidermoid carcinoma cells, MDA-MB-231 human breast carcinoma cells, U118MG glioma carcinoma cells and H1975 human lung adenocarcinoma cells. As shown in Figure 8, JC-PS1 exhibited significant anti-proliferation activities, and a dose-dependent behavior was observed for proliferation inhibition for A431, MDA-MB-231, U118MG and H1975 cells while it was not observed for PANC-1 cells. When the concentration of JC-PS1 was as low as 0.16 mg/mL, the cell viability decreased significantly (*p* < 0.001) for PANC-1, A431, U118MG and MDA-MB-231cells while *p* < 0.05 for H 1975 cells, indicating that JC-PS1 was more sensitive to PANC-1, A431, U118MG and MDA-MB-231 cells than to H 1975 cells. When JC-PS1 was at the concentration of 2.5 mg/mL (8.928 nM), the inhibitory effects reached to maximum and the cell viability of PANC-1, A431, MDA-MB-231, U118MG and H1975 cells was 35.95%, 6.75%, 27.31%, 18.65% and 10.96% while the cell viability was 26.54%, 8.71%, 10.67%, 75.16% and 7.91% for the positive control (DOX) at 0.1 mg/mL (172.41 nM). This suggested that, compared with DOX, JC-PS1 was a potential antitumor agent and had better inhibitory effects on U118 MG cells. In addition, the IC_50_ values of JC-PS1 for PANC-1, A431, MDA-MB-231, U118MG and H1975 cells were 296.8, 477.9, 657.4, 686.7, 862.1 μg/mL, respectively. Additionally, JC-PS1 showed no toxicity on normal cells (RAW264.7 cells) in Figure S3, indicating that JC-PS1 was potentially a safe and effective therapeutic agent for tumors.

In addition, we also isolated and purified a polysaccharide (JC-PS2) from the water fraction of DEAE 52 Cellulose column. However, JC-PS2 showed no anti-proliferation activities. Comparing the difference between JC-PS1 and JC-PS2 in structure, we found that JC-PS1 was characterized by a backbone of →5)-α-Ara*f*-(1→ and →3,5)-α-Ara*f*-(1→, while the backbone of JC-PS2 was mainly composed of →4)-α-Glc*p*-(1→ (Table S1, Figure S4). This implied that the backbone of →5)-α-Ara*f*-(1→ and →3,5)-α-Ara*f*-(1→ might affect the anti-proliferation activities, which was in accordance with the reported literature [9].

## 3. Materials and Methods

### 3.1. Materials and Reagents

The twigs and leaves of *J. convallium* were collected in May, 2017 in Lhasa, Tibet, PR China, and were identified by associate Prof. Ji De (College of Science, Tibet University). The voucher specimen (JC201705) was kept in the School of Pharmacy, Fudan University, Shanghai, PR China. In addition, then the plants were crushed into powder by a disintegrator.

DEAE-cellulose 52 was purchased from Whatman (Maidstone, England), and Sephacryl^TM^ S-300 High Resolution from Amersham Biosciences (Uppsala, Sweden). Trichloroacetic acid (TCA), trifluoroacetic acid (TFA), sodium borohydride and iodomethane were purchased from Sino-pharm Chemical Reagent Co. Ltd. (Shanghai, China). NaBH4, D_2_O and anhydrous DMSO were purchased from Adamas-beta. Co. Ltd. (Shanghai, China). Monosaccharide standards (glucuronic acid, galacturonic acid, glucose, galactose, mannose, arabinose, ribose, xylose, and rhamnose) and N-cyclohexyl-N’-(2-morpholinoethyl) carbodiimide methyl-*p*-toluenesulfonate (CMC) and 1-phenyl-3-methyl-5-pyrazolone (PMP) were purchased from Sigma-Aldrich (St. Louis, USA). All other reagents were of the highest available quality.

### 3.2. Extraction, Isolation, and Purification of JC-PS1

The dried crude powder of *J. convallium* (2.1 kg) was firstly refluxing extracted by 95% ethanol (10 L) three times in order to remove the alcohol-soluble pigments and small organic molecules. Then the residues were dried and extracted with distilled water at a ratio of 1:10 (*w*/*v*) at 100 °C three times (2 h for each time). Then the supernatant was combined and concentrated into one-fifth of the original volume, followed by precipitation by adding four-fold volume of ethanol and standing at 4 °C overnight. The precipitate was collected by centrifugation (3500 rpm, 20 min), dissolved in distilled water and deprotionized with the trichloroacetic acid method. Briefly, one portion of the dissolved extract and one portion of 20% trichloroacetic acid (*w*/*v*) were mixed and treated at 4 °C overnight to remove proteins three times. In addition, then the supernatant was neutralized with 1 M NaOH after centrifugation, and finally was concentrated and freeze-dried to obtain the crude polysaccharides (JCP, 133.6 g).

JCP (80 g) were dissolved in distilled water and then applied to a DEAE-52 Cellulose column (12 cm × 100 cm) which was equilibrated with ultrapure water and eluted by water and 0.1 M NaCl solution sequentially at a flow rate of 25 mL/min. The fraction which was eluted by 0.1 M NaCl solution was collected, dialyzed, and freeze-dried to obtain the JC-0.1 M (16.06 g). In addition, the JC-0.1 M (2 g) was then further purified by Sephacryl S-300 gel filtration column (2.5 cm × 80 cm), eluted with ultrapure water at a flow rate of 0.5 mL/min to obtain a polysaccharide named JC-PS1 (500 mg).

### 3.3. Homogeneity and Relative Molecular Weight Analysis

The homogeneity of JC-PS1 was determined by high performance gel permeation chromatography (HPGPC) on an Agilent 1200 HPLC system equipped with an evaporative light scattering detector (ELSD) and a Tskgel GMPWXL column (7.8 mm × 30 cm, TOSOH company, Japan). In addition, the column was eluted with ultrapure water at a flow rate of 0.8 mL/min and the temperature of 30 °C.

The relative molecular weight of JC-PS1 was also determined by HPLC-ELSD with the standard curve method. A set of dextrans with different molecular weights were taken as standards, and the molecular weight was calculated according to standard curve established by standards.

### 3.4. Analysis of Chemical Composition

The sugar content of JC-PS1 was determined by the phenol sulfuric acid method [30] with glucose as the reference. Then Taking the GalA as reference, the *m*-hydroxybiphenyl method [31] was adopted to analyze the uronic acid of JC-PS1. In addition, the protein content was analyzed by Bradford’s method [32], with bovine serum albumin as the standard.

### 3.5. Determination of Monosaccharide Composition

Monosaccharide composition of JC-PS1 was analyzed on LC-MS by the method of 1-phenyl-3-methyl-5-pyrazolone (PMP) pre-column derivatization [33]. Briefly, JC-PS1 (2 mg) was completely hydrolyzed by trifluoroacetic acid (TFA). In addition, the hydrolysate was further dissolved in ammonium hydroxide and reacted with 0.5 M PMP-CH_3_OH solution (0.5 mL) at 70 °C for 100 min to be labeled with PMP. In addition, then the reaction products were extracted with chloroform (*v*/*v* = 3:1) three times to remove the remaining PMP. The upper aqueous phase was filtered by 0.45 μm membrane for analysis. Finally, the sample (10 μL) was applied to LC-MS (Dionex Ultimate 3000, Thermo Fisher Scientific Co Ltd., USA) equipped with YMC-Triart C18 (150 mm × 21 mm) column, and eluted with the mixture of 0.02 M ammonium carbonate solution and acetonitrile (17:83, pH = 6.8) at 30 °C with a flow rate of 0.3 mL/min. The same procedure was applied to the monosaccharide standards.

### 3.6. Infrared Spectroscopy (IR) Spectrum Analysis

The IR spectrum of JC-PS1 was conducted on a FT-IR spectrometer (Nicolet iS5, Thermo Fisher Scientific Co Ltd., Waltham, MA, USA). JC-PS1 (1 mg) was mixed together with potassium bromide and pressed into pellets. Then the pellets were scanned from 4000 cm^−1^ to 400 cm^−1^.

### 3.7. Methylation Analysis

JC-PS1 was dissolved with distilled water and treated with CMC for 3 h. In addition, then the uronic acid in JC-PS1 was reduced by NaBH_4_. The reduced JC-PS1 was methylated based on the reported method [34] with some modification. First of all, the sodium metacylsulfonyl methyl (SMSM) was prepared by continuously adding NaH (5 g), which had been washed previously with petroleum ether, in DMSO (50 mL) at 50–60 °C with magnetic stirring for 4 h. Then JC-PS1 (15 mg) was dissolved in DMSO (15 mL) and the SMSM (5 mL) was dripped slowly under ultrasonic treatment. The reaction products were treated with ultrasonic for another 15 min, followed by slowly adding methyl iodide in ice bath. The methylated polysaccharide was extracted from chloroform after dialysis for 24 h, and was then treated with the same procedure another two times until the 3200–3700 cm^−1^ O-H band in IR spectrum disappeared. Finally, the methylated polysaccharide was hydrolyzed completely by being reacted with 2 M TFA at 100 °C for 6 h. and the hydrolysates were reduced with NaBD_4_ and acetylated with the mixture of acetic anhydride and pyridine (*v*/*v* =1:1). Finally, the reaction product was analyzed by GC-MS (QP2010, Shimadzu Co Ltd., Kyoto, Japan).

### 3.8. NMR Analysis

To further acquire the structural information of JC-PS1, NMR spectroscopy (DRX-600, Bruker Corporation, Germany) was conducted with D_2_O as the solvent at 25 °C, including ^13^C NMR, ^1^H NMR, HSQC, HMBC and COSY. The chemical shifts were expressed in ppm with the HOD signal fixed at 4.79 ppm.

### 3.9. AFM Analysis

An AFM (Multimode NanoscopeIIIa, Bruker Corporation, Germany) was used to identify the surface topography of JC-PS1. In brief, JC-PS1 was diluted in distilled water to the final concentration of 10 μg/mL. 200 μL of the sample was dropped onto the freshly cleaved mica and dried under the infrared lamp before imaging. In addition, the AFM image was conducted by the Nanoscope software.

### 3.10. Anti-Proliferation Assay of JC-PS1

The anti-proliferation assay of JC-PS1 was conducted by the MTT method [35]. Five tumor cell lines, including PANC-1 pancreatic carcinoma cells, A431 human epidermoid carcinoma cells, MDA-MB-231 human breast carcinoma cells, U118MG glioma carcinoma cells and H1975 human lung adenocarcinoma cells, were seeded at a density of 1.0 × 10^4^ cells/well on the microplate with 96 wells. The 96-well microplate was incubated at 37 °C for 4 h to obtain cell attachment. In addition, then 100 μL of pure fresh medium was added and incubated at 37 °C for 48 h as the blank control group with fresh medium containing JC-PS1 (0.16–2.5 mg/mL) and doxorubicin (DOX, 0.1 mg/mL) as experiment group and positive control group, respectively. The medium was removed and 50 μL of methylene blue was added to each well. In addition, finally absorbance at 570 nm was measured by a microplate. Each sample was measured in triplicate. The cell viability was calculated by OD 570 nm _sample_/OD 570 nm _control_.

### 3.11. Statistical Analysis

All data were expressed as the means ± SD and analyzed by Graphpad Prism Program version 6.02. The *p*-value of the difference between groups was measured using the Student’s *t*-test for comparison or one-way ANOVA for multiple comparisons. *p* < 0.05 was considered statistically significant.

## 4. Conclusions

Small molecules, such as derivatives of podophyllotoxin and essential oils from *Juniperus* species had been reported to show significant cytotoxicity against tumor cells. The polysaccharide JC-PS1 firstly reported in this study revealed the antitumor potential of the polysaccharides from the plants of this genus. JC-PS1 was characterized by the backbone of →5)-α-Ara*f*-(1→ and →3,5)-α-Ara*f*-(1→ and three branches linked to the O-3 position of →3,5)-α-Ara*f*-(1→ in the molar ratio of 2:1:1, including β-Gal*p*A-(1→3)-β-Gal*p*-(1→, α-Ara*f*-(1→3)-α-Rha*p*-(1→ and α-Ara*f*-(1→3)-β-Gal*p*-(1→. In the antitumor activity test, JC-PS1 exhibited the broad spectrum of significant antitumor cell proliferation activities against human carcinoma cells and no toxicity on normal cells, indicating that JC-PS1 is potentially a safe and effective therapeutic agent in the treatment of cancer. In addition, the underlying mechanisms and structure-activity relationship remain to be further studied and discussed.

## Figures and Tables

**Table 1:** Alditol acetate derivatives from the methylated JC-PS1

No	Methylations Sugars	Linkage	Molar Ratios	MS Fragments
1	2,3-Me2-Ara*f*	→5)-Ara*f*-(1→	3.9	59,71,87,102,118,129,189
2	2,3,5-Me3-Ara*f*	Ara*f*-(1→	1.0	59,71,87,102,118,145,161,162
3	2-Me-Ara*f*	→3,5)-Ara*f*-(1→	2.1	59,74,85,99,118,127,159,173, 261
4	2,4,6-Me3-Gal*p*	→3)-Gal*p*-(1→	1.5	59,74,87,101,118,129,161,234,277
5	2,4-Me2-Rha*p*	→3)-Rha*p*-(1→	0.5	59,72,89,101,118,131,234,247
6	2,3,4,6-Me4-Gal*p*	Gal*p*-(1→	1.0	59,71,87,102,118,129,145,161,205

**Table 2:** NMR signal assignment of JC-PS1

Residues		C1/H1	C2/H2	C3/H3	C4/H4	C5/H5	C6/H6
A	→5)-α-Ara*f*-(1→	106.93 5.02	80.47 4.06	76.21 3.93	81.55 4.14	66.41 3.73	- -
B	α-Ara*f*-(1→	106.51 5.10	80.95 4.08	76.08 3.89	77.99 4.05	60.45 3.65	- -
C	→3,5)-α-Ara*f*-(1→	106.05 5.17	80.39 4.24	83.33 4.01	79.83 4.22	65.80 3.87	- -
D	→3)-β-Gal*p*-(1→	98.96 4.96	75.88 3.76	78.51 3.59	73.55 3.82	68.64 3.93	60.61 3.65
E	→3)-α-Rha*p*-(1→	98.00 5.02	80.37 4.21	81.21 3.82	66.10 3.73	66.05 3.70	15.96 1.18
F	β-Gal*p*A-(1→	102.41 4.42	68.55 3.29	70.73 3.49	71.46 3.63	74.43 3.48	175.04 -

## Supplementary Materials

The following are available online, Figure S1: The IR spectrum of JC-PS1, Figure S2: ^1^H-^1^H COSY spectrum of JC-PS1, Figure S3: Cytotoxicity of JC-PS1 on RAW264.7 cells. Figure S4: The repeating unit of JC-PS2. Table S1: Alditol acetate derivatives from the methylated JC-PS2.
