# Supplementary material for: Structural Characterization and Anti-Proliferation Activities Against Tumor Cells of an Arabinogalactan from Juniperus convallium
Source: Molecules. 2019 May 14;24(10):1850. doi: 10.3390/molecules24101850 (PMC6571648; doi:10.3390/molecules24101850)
Supplement: Supplementary file 1 [file molecules-24-01850-s001.pdf]

Structural characterization and anti-proliferation activities against tumor cells of an arabinogalactan from *Juniperus convallium*

Beibei Li [1], Mengxue Dong [1], Ji De [2], Li Ye [1], Daofeng Chen [1] and Yan Lu [1,*]

[1] School of Pharmacy, Fudan University, Shanghai 201203, People's Republic of China
[2] College of Science, Tibet University, Lhasa 850000, Tibet, People's Republic of China

* Correspondence: luyan@fudan.edu.cn; Tel.: +86-021-51980157 (Y.L.)

Figure S1. The IR spectrum of JC-PS1

Figure S2. ^{1}H-^{1}H COSY spectrum of JC-PS1.

Figure S3. Cytotoxicity of JC-PS1 on RAW264.7 cells. The data were represented as mean ± SD (n=3).

Figure S4. The repeating unit of JC-PS2.

Table S1. Alditol acetate derivatives from the methylated JC-PS2.

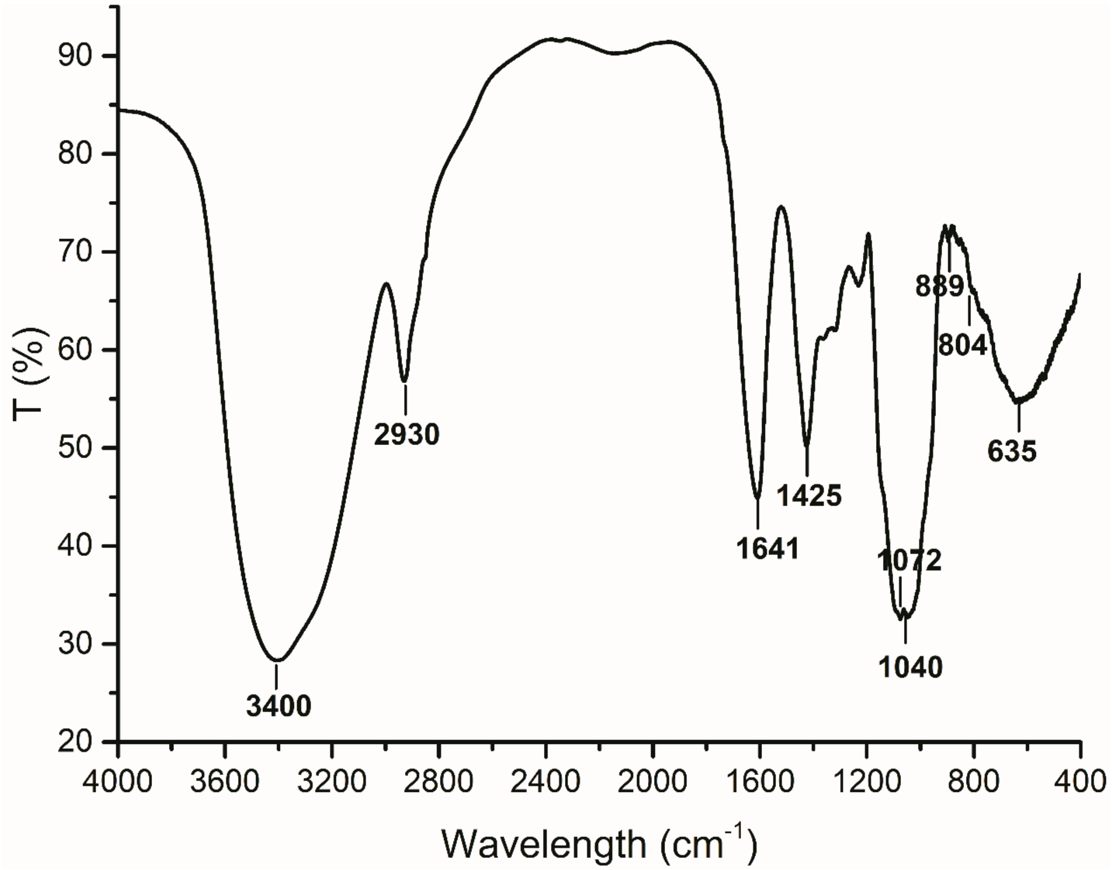

Figure S1. The IR spectrum of JC-PS1

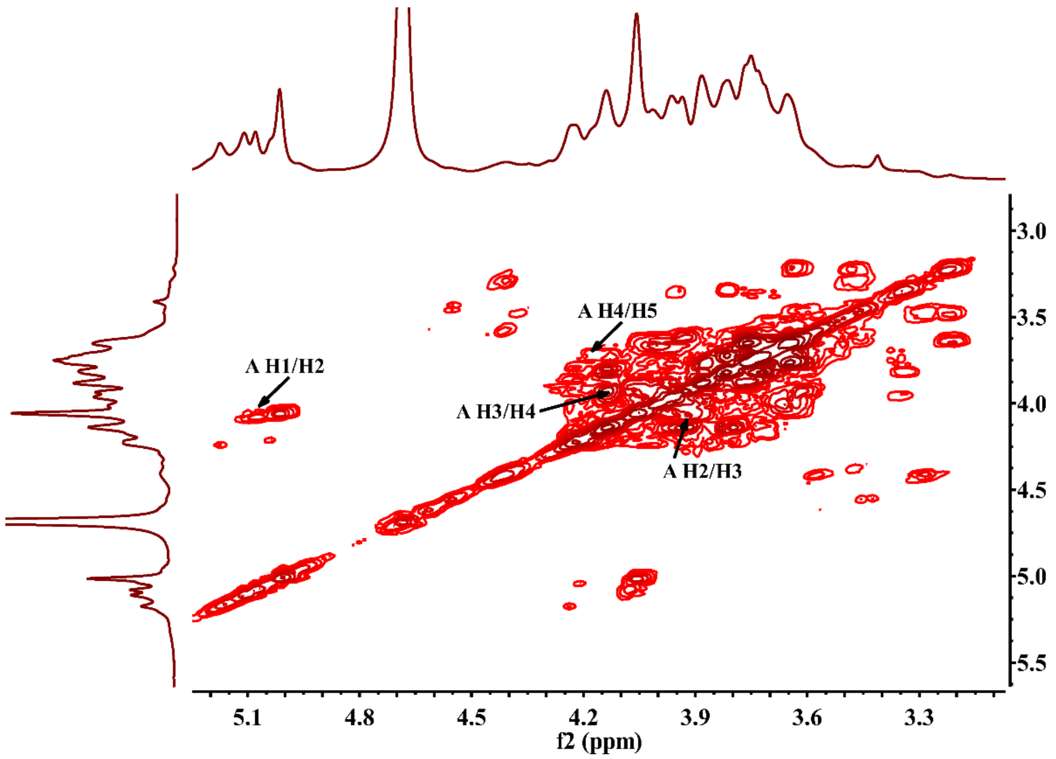

Figure S2. ^{1}H-^{1}H COSY spectrum of JC-PS1.

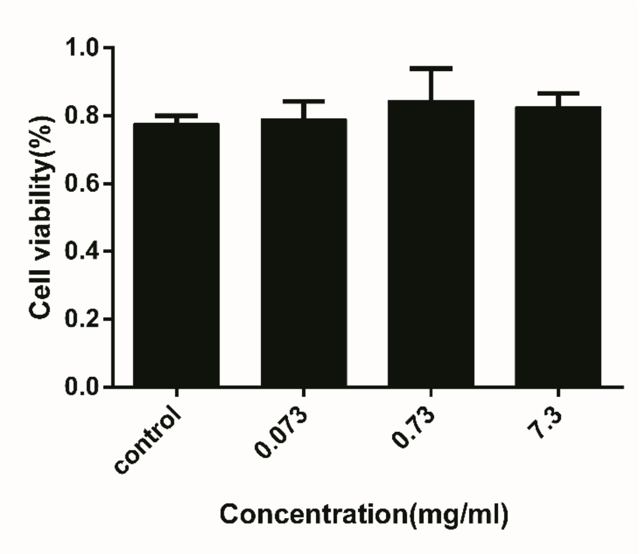

Figure S3. Cytotoxicity of JC-PS1 on RAW264.7 cells. The data were represented as mean ±SD (n=3).

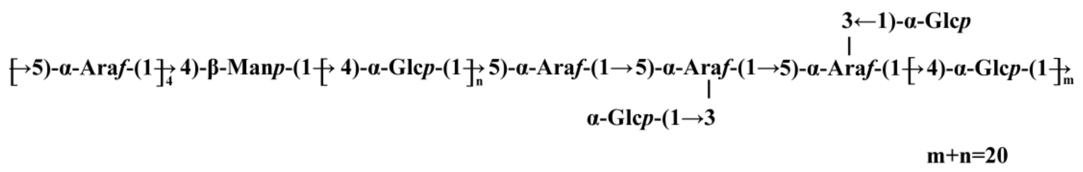

Figure S4. The repeating unit of JC-PS2.

Table S1. Alditol acetate derivatives from the methylated JC-PS2

No	Methylations sugars	Linkage	Molar ratios	MS Fragments
1	2,3,6-Me3-Glc*p*	→4)-Glc*p*-(1→	30.1	71,87,99,101,113,117,129,131,173,233
2	2,3,4,6-Me4-Glc*p*	Glc*p*-(1→	3.1	71,87,101,117,129,145,161,205
3	2,3,6-Me3-Man*p*	→4)-Man*p*-(1→	1.1	71,87,99,101,113,117,129,131,161,233
4	2-Me-Ara*f*	→3,5)-Ara*f*-(1→	3.5	71,85,99,117,127,159,201,261
5	2,3-Me2-Ara*f*	→5)-Ara*f*-(1→	10.4	71,87,101,117,129,161,189
